# X Chromosome Inactivation during *Drosophila* Spermatogenesis

**DOI:** 10.1371/journal.pbio.0050273

**Published:** 2007-10-09

**Authors:** Winfried Hense, John F Baines, John Parsch

**Affiliations:** Department of Biology II, University of Munich, Planegg-Martinsried, Germany; Duke University, United States of America

## Abstract

Genes with male- and testis-enriched expression are under-represented on the Drosophila melanogaster X chromosome. There is also an excess of retrotransposed genes, many of which are expressed in testis, that have “escaped” the X chromosome and moved to the autosomes. It has been proposed that inactivation of the X chromosome during spermatogenesis contributes to these patterns: genes with a beneficial function late in spermatogenesis should be selectively favored to be autosomal in order to avoid inactivation. However, conclusive evidence for X inactivation in the male germline has been lacking. To test for such inactivation, we used a transgenic construct in which expression of a *lacZ* reporter gene was driven by the promoter sequence of the autosomal, testis-specific *ocnus* gene. Autosomal insertions of this transgene showed the expected pattern of male- and testis-specific expression. X-linked insertions, in contrast, showed only very low levels of reporter gene expression. Thus, we find that X linkage inhibits the activity of a testis-specific promoter. We obtained the same result using a vector in which the transgene was flanked by chromosomal insulator sequences. These results are consistent with global inactivation of the X chromosome in the male germline and support a selective explanation for X chromosome avoidance of genes with beneficial effects late in spermatogenesis.

## Author Summary

During spermatogenesis, the X chromosome is inactivated in the male germline (sperm cells), thereby silencing, or inactivating, genes residing on the X chromosome. X chromosome silencing is thought to be common among species with XY sex determination and has important implications for genome evolution. For example, genes with increased expression in the male tend to be under-represented on the X chromosome, and many testes-specific genes have been “retrotransposed,” or moved, from the sex to autosomal chromosomes. However, compelling evidence for X chromosome inactivation in the fruit fly *Drosophila* has been lacking. Here, we used a transgenic technique to test for male germline X inactivation in this important model organism. We randomly inserted a “reporter gene” whose expression requires a regulatory element for an autosomal testis-specific gene into multiple autosomal and X-chromosomal locations. We found that autosomal insertions of the reporter gene have significantly higher expression in the male germline than X-linked insertions. This pattern holds for two different transgenes with nearly 50 independent insertions, providing strong evidence for X chromosome inactivation during spermatogenesis. The silencing of X-linked gene expression in the male germline may contribute to the observed paucity of male-expressed genes on the X chromosome and the excess of retrotransposed genes that have moved from the X chromosome to the autosomes.

## Abstract 3

Genes expressed in the *Drosophila* testes are under-represented on the X chromosome to avoid the global inactivation of the X chromosome that occurs in the male germ line.

## Introduction

Sex chromosomes, such as the X and Y chromosomes of *Drosophila*, are thought to have evolved from a pair of homologous autosomes that lost their ability to recombine with each other [1,2]. Over evolutionary time, the sex chromosome that is present only in the heterogametic sex (the Y in *Drosophila* and mammals) tends to degenerate, losing most of its gene complement and accumulating transposable elements [3–6]. The X chromosome, which is still able to recombine within the homogametic sex, maintains a fully functional complement of genes and resembles an autosome in its size, cytogenetic appearance, repetitive element content, and gene density. Recent genomic studies, however, have revealed a number of more subtle differences in gene content, expression pattern, and molecular evolution between the X chromosome and the autosomes [7].

One pattern that has emerged from the genomic analysis of Drosophila melanogaster is that there is a significant excess of gene duplications in which a new autosomal gene has arisen from an X-linked parental gene through retrotransposition [8]. Most of these new autosomal genes appear to be functional and are expressed in testis [8]. Several of these genes that have been studied in detail show evidence of adaptive evolution and/or functional diversification [8–11]. Another pattern that has emerged from functional genomic studies is that genes with male-enriched expression are under-represented on the X chromosome [12,13]. For example, about 19% of all D. melanogaster genes reside on the X chromosome, but only 11% of the genes with a 2-fold or greater male bias in expression are X-linked [14]. Furthermore, the male-biased genes that are X-linked tend to show less sex bias in their expression than those that are autosomal [15].

A number of hypotheses have been put forth to explain the above observations [16–18]. To explain the large excess of retrotransposed genes that have “escaped” the X chromosome, Betrán et al. [8] proposed the X inactivation hypothesis, which posits that genes with a beneficial effect late in spermatogenesis are selectively favored to be autosomally located. Otherwise, their expression would be prevented by male germline X inactivation, which is supposed to occur early in spermatogenesis when autosomal genes are still actively transcribed. Early X inactivation could also explain the paucity of genes with male-biased expression on the X chromosome: if X-linked genes cannot be expressed in the later stages of spermatogenesis, then one would expect to see fewer X-linked genes with enriched expression in adult males. In particular, this should be true for genes expressed in the male germline and those encoding sperm proteins, which has been observed [12,19].

Male germline X inactivation, however, cannot completely explain the observations. For instance, male-biased genes that are expressed only in somatic cells, where X inactivation does not occur, are also significantly under-represented on the X chromosome [12,20]. An alternative explanation that accommodates this observation invokes sexual antagonism, that is, evolutionary conflict between males and females. The fixation probability of an X-linked, sexually antagonistic mutation is expected to differ from that of an autosomal one, with the direction of this difference depending on the dominance coefficient [21,22]. If the antagonistic effects are (at least partly) dominant, then female-beneficial/male-harmful mutations will accumulate on the X chromosome, while male-beneficial/female-harmful mutations will be removed from the X. This is because the X chromosome spends two-thirds of its evolutionary history in females and, thus, is more often under selection in the background of this sex. Since genes with sex-biased expression may be prime targets for sexually antagonistic mutations, the above scenario could lead to an excess of female-biased genes and a paucity of male-biased genes on the X [13], resulting in “feminization” or “demasculinization” of this chromosome [12].

A hypothesis that combines the concepts of sexual antagonism and X inactivation was proposed by Wu and Xu [23]. This hypothesis, termed SAXI (sexually antagonistic X inactivation), suggests that natural selection has favored the movement of sexually antagonistic X-linked genes whose expression is beneficial to males to the autosomes, leaving those beneficial to females on the X. Over evolutionary time, the accumulation of female-beneficial/male-harmful genes on the X leads to selection for X inactivation in the male germline, particularly during the later stages of spermatogenesis where the effects of sexual antagonism are expected to be greatest [23]. The hypotheses of Betrán et al. [8] and Wu and Xu [23] assume that the X chromosome becomes inactive before the autosomes during spermatogenesis. This phenomenon has been established in mammals and nematodes [24–26]. However, the evidence for male germline X inactivation in *Drosophila* has been equivocal. Lifschytz and Lindsley [27] cited cytological observations and genetic experiments to argue that X inactivation during spermatogenesis was common to most animal species with heterogametic males, including D. melanogaster. However, similar evidence was used to argue against X inactivation in *Drosophila* [28]. A later study of the expression of sperm-specific proteins in transgenic *Drosophila* provided experimental support for X inactivation [29]. Here the authors used a testis-specific promoter to drive the expression of altered forms of β-tubulins in the male germline and noted that X-linked inserts of the constructs showed reduced expression relative to autosomal inserts. Although this result was consistent with X inactivation, there were some limitations. For instance, the sample sizes were small for each of the expression constructs, with only one or two X-linked inserts per construct. Furthermore, the expression level of the genes was only roughly estimated from protein abundance on electrophoresis gels.

A more recent experimental study failed to find support for male germline X inactivation in *Drosophila* [30]. These authors examined the expression and intracellular location of the MLE protein (encoded by *maleless*), as well as the acetylation pattern of histone H4, in male germline cells. Although MLE is known to be involved in X chromosome hypertranscription in somatic cells, presumably through the recruitment of histone acetylation factors [31,32], it does not associate specifically with the X chromosome in male germ cells. Furthermore, H4 acetylation at lysine 16, which is thought to be a reliable marker for active transcription, was observed equally on the X chromosome and the autosomes. Thus, there was no evidence for dosage compensation or X inactivation in the male germline. However, it is not necessary that these two processes occur through the same mechanism, or that they rely on the same proteins required for somatic cell dosage compensation. Indeed, a microarray analysis of germline gene expression indicated that dosage compensation does occur in the male germline [33]. Because these microarray experiments used reproductive tissues that contained somatic cells and germline cells from all stages of gametogenesis, they could not directly address the issue of early X inactivation. However, the fact that most X-linked genes showed similar levels of expression in both male and female reproductive tissues suggests that, if X chromosome inactivation does occur in the male germline, it does not have a large effect on the global pattern of sex-biased gene expression.

In this study, we perform a more rigorous experimental test for X inactivation in the male germline. Using a transgenic construct in which the expression of a reporter gene is driven by the promoter of the autosomal, testis-specific *ocnus (ocn)* gene, we show that autosomal inserts are expressed specifically in males and in testis. X-linked inserts, in contrast, show greatly reduced levels of expression. These results hold for a large sample of independent insertions and for two different transformation vectors and, thus, provide strong support for inactivation of the X chromosome during *Drosophila* spermatogenesis.

## Results

### Identification and Functional Analysis of the *ocn* Promoter

**Figure 1:**
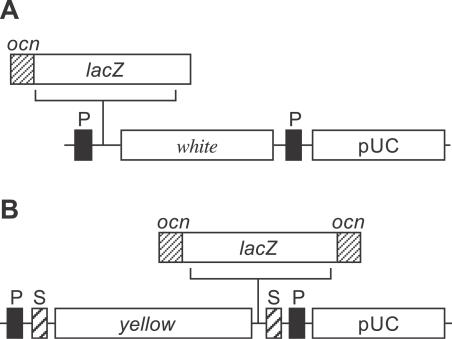
Schematic Diagram of the *ocn*-*lacZ* Expression Constructs (A) The p*P[wFl-ocn-lacZ]* vector. The *ocn* promoter fused to the *lacZ* open reading frame was inserted into the p*P[wFl]* transformation vector, which contains the *white* gene as a selectable marker. The boundaries of the DNA inserted into the *Drosophila* genome are indicated by “P”. The portion of the plasmid used for replication in E. coli is labeled “pUC”. (B) The p*P[YEStes-lacZ]* vector. The *ocn* promoter and 3′ UTR were fused to respective ends of the *lacZ* open reading frame and inserted into the YES transformation vector. Binding sites for the Suppressor of Hairy-wing protein, which functions as a chromosomal insulator, are labeled “S”.

**Figure 2:**
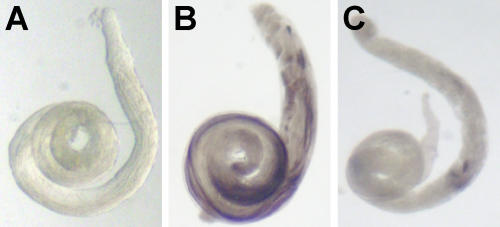
Reporter Gene Expression in Testis Testes were dissected and incubated with S-GAL, which forms a black precipitate in the presence of β-galactosidase. Shown are testes from *y w* males (negative control) (A), *y w* males with an autosomal insertion of *P[wFl-ocn-lacZ]* (B), and *y w* males with an X-linked insertion of *P[wFl-ocn-lacZ]* (C). Staining was performed in parallel for the same length of time. The strongest signal is in the proximal region of the autosomal-insert testis. Note that weak staining is visible in the proximal region of the X-insert testis.

The *ocn* gene is expressed specifically in testis and encodes a protein abundant in mature sperm [19,34]. It is part of a cluster of three tandemly duplicated genes on chromosome arm 3R that are present in all species of the D. melanogaster species subgroup and shares greatest homology to the neighboring *janusB (janB)* gene, which is also expressed in testis. Although *ocn* lies only 250 bp distal to *janB,* it produces a unique transcript that does not overlap with that of *janB* [34]. The first half of the *janB*-*ocn* intergenic region is highly diverged among species of the D. melanogaster subgroup and cannot be aligned unambiguously. However, the portion just upstream of the *ocn* start codon is well conserved, suggesting that it has regulatory function (Figure S1). We refer to this region as the *ocn* promoter. To test its ability to drive tissue-specific gene expression, we fused it to the open reading frame of the *Escherichia coli lacZ* gene, which encodes the enzyme β-galactosidase (Figure 1A). Transgenic flies with autosomal insertions of *P[wFl-ocn-lacZ]* showed reporter gene expression specifically in testis, as expected (Figure 2).

### Comparison of Autosomal and X-Linked Insertions

Overall, we obtained 15 independent autosomal insertions of *P[wFl-ocn-lacZ]*. The mean β-galactosidase activity in adult males was 8.67 units, while that in adult females was 0.34 units. The difference between the sexes was highly significant (Mann-Whitney U test, *p* < 0.001). The mean β-galactosidase activity of gonadectomized males was 0.24 units, which was significantly less than whole males (Mann-Whitney U test, *p* < 0.01).

**Figure 3:**
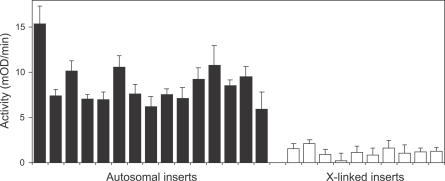
Average β-Galactosidase Activity of Adult Male Flies with Autosomal (Solid Bars) or X-Linked (Open Bars) Insertions of *P[wFl-ocn-lacZ]* Each bar represents a different transformed line with a unique, independent transgene insertion. Error bars indicate the standard error of the mean, calculated from the variance among all replicate measurements within each independent insertion line.

If the X chromosome is inactivated before the autosomes during spermatogenesis, then one would expect transgenic lines with X-linked insertions of *P[wFl-ocn-lacZ]* to show lower levels of reporter gene expression than those with autosomal insertions. This is indeed what we observed. In total, we obtained ten independent X-linked insertions of *P[wFl-ocn-lacZ]*. All of these lines showed reduced β-galactosidase activity in adult males relative to the autosomal-insertion lines (Figures 2 and 3). On average, the activity difference between autosomal and X-linked insertions was 7-fold (8.67 versus 1.19 units), and the difference between the two groups was highly significant (Mann-Whitney U test*, p* < 0.001). Although β-galactosidase activity was very low for the X-linked insertions, it was significantly greater than zero. Assuming a normal distribution of activity among the X-insertion lines, the 95% confidence interval was 0.82–1.56 units. Five of the autosomal insertion lines (the last five in Figure 3) were obtained through the re-mobilization of X-linked inserts (see Materials and Methods), demonstrating that the reduction in expression was not caused by undesired sequence changes in the *ocn* promoter or *lacZ* coding sequence, but instead was a direct result of X linkage.

**Figure 4:**
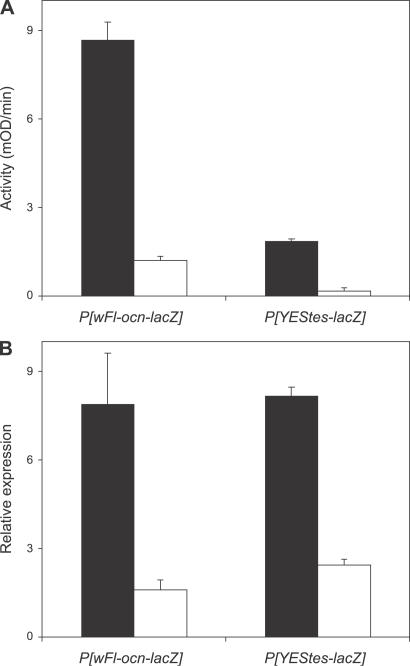
Expression Levels of Autosomal (Solid Bars) and X-Linked (Open Bars) Insertions of the Two *ocn-lacZ* Transformation Vectors Shown in Figure 1 (A) Average β-galactosidase activity of adult males. (B) Relative expression measured by qRT-PCR. Transcript abundance was standardized to that of the ribosomal protein gene *RpL32* and is given in arbitrary units. Error bars indicate the standard error of the mean, calculated from the variance among the means of the independent insertion lines.

Because the assays of β-galactosidase activity measure expression at the level of protein abundance, it is possible that they do not reflect underlying levels of transcription. To test this possibility, we performed quantitative reverse-transcription PCR (qRT-PCR) to estimate the relative transcript abundance of a subset of eight transformed lines, including four with autosomal and four with X-linked inserts. The autosomal inserts had significantly higher transgene expression at the level of mRNA (Mann-Whitney U test, *p* = 0.02), with the relative expression difference being 5-fold (Figure 4B), which corresponds well to the observed difference in β-galactosidase activity and suggests that the enzymatic assays provide a reliable estimate of expression.

### Effect of Chromosomal Insulator Sequences

To test if the reduced expression of the X-linked *ocn*-*lacZ* transgenes could be attributed to the presence of localized transcriptional repressors bound to the X chromosome, we performed additional experiments using the *P[YEStes-lacZ]* transformation vector (Figure 1B), which contains binding sites for the protein encoded by *suppressor of Hairy-wing*. These binding sites flank the inserted transgene and serve to insulate it from the effects of external transcriptional regulators [35]. We obtained 12 independent autosomal insertions of *P[YEStes-lacZ],* and these lines showed male- and testis-specific expression of the *lacZ* reporter gene. The mean β-galactosidase activity in adult males was 1.84 units, which was significantly greater than that of adult females (mean = 0.42; Mann-Whitney U test, *p* < 0.001) or gonadectomized males (mean = 0.22; Mann-Whitney U test, *p* < 0.001).

We also obtained ten independent insertions of *P[YEStes-lacZ]* on the X chromosome. Adult males of these lines had a mean β-galactosidase activity of 0.17 units, which differed significantly from the autosomal-insert lines (Mann-Whitney U test*, p* < 0.001), but did not differ significantly from zero (95% confidence interval = −0.09–0.43). The reduction in reporter β-galactosidase activity caused by X linkage was more than 10-fold (Figure 4A). We also assayed expression at the level of transcript abundance by performing qRT-PCR on a subset of eight transformed lines (four with autosomal and four with X-linked inserts). Again, the X chromosome insertion lines showed significantly less transgene expression than the autosomal insertion lines (Mann-Whitney U test*, p* = 0.02). The reduction in reporter gene expression measured by qRT-PCR was 3.4-fold (Figure 4B). Thus, the presence of the chromosomal insulator sequences did not alleviate transcriptional repression of the X-linked transgenes.

For adult males with autosomal insertions, the coefficient of variation (CV) for β-galactosidase activity was lower among the *P[YEStes-lacZ]* transformed lines (CV = 0.16) than among the *P[wFl-ocn-lacZ]* transformed lines (CV = 0.28). A more pronounced difference was seen at the level of mRNA abundance, where the CVs for *P[YEStes-lacZ]* and *P[wFl-ocn-lacZ]* transformants were 0.07 and 0.44, respectively. This suggests that the insulator sequences successfully reduced position effect variation caused by the chromosomal context of the insertion. The *P[YEStes-lacZ]* transformants, however, showed significantly less β-galactosidase activity than the *P[wFl-ocn-lacZ]* transformants (Mann-Whitney U test, *p* < 0.001; Figure 4A). Interestingly, this difference was not detectable at the level of mRNA abundance (Figure 4B), which suggests additional, post-transcriptional regulation of the *P[YEStes-lacZ]* transgenes.

## Discussion

Although a number of hypotheses regarding genome and sex chromosome evolution assume that the *Drosophila* X chromosome becomes transcriptionally inactive before the autosomes during spermatogenesis, little direct evidence for this scenario has been reported. Our experimental results indicate that X chromosome inactivation does occur in *Drosophila* and that it can have a considerable effect on gene expression in the male germline. In total, we examined 27 autosomal and 20 X-linked insertions of a testis-specific reporter gene in two different transformation vectors. In all cases, transformed lines with autosomal insertions showed significantly greater transgene expression than their X-linked counterparts, with the differences in expression ranging from 3.4- to 10-fold. The consistency of these results across a large number of independent insertions suggests that this transcriptional inactivity is a global property of the X chromosome. The fact that we observe the same pattern when using a vector that insulates the transgene from external transcriptional regulators further suggests that inactivation of the X chromosome in the male germline occurs through a major structural change, rather than by the binding of localized transcriptional repressors.

**Figure 5:**
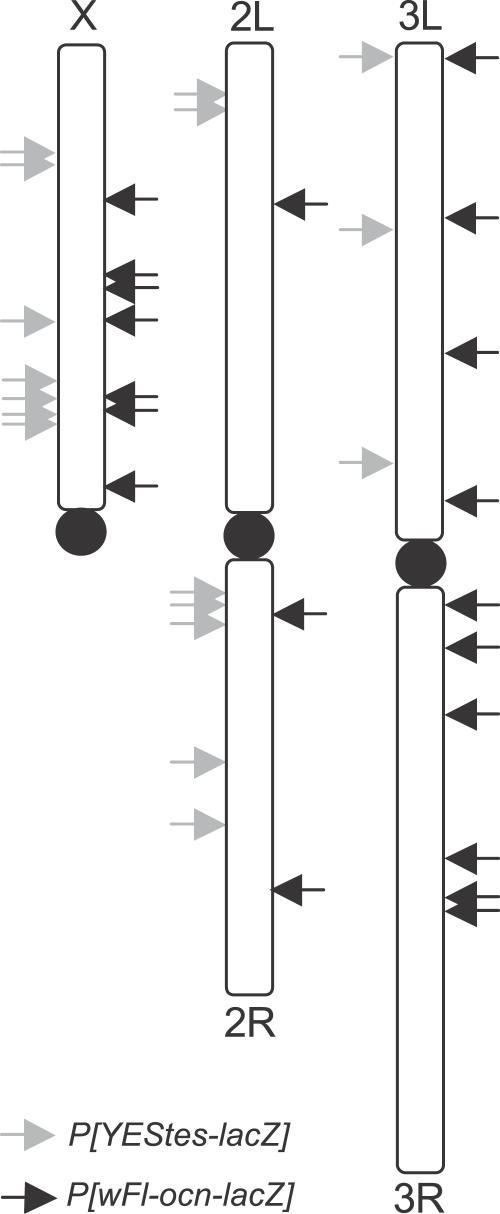
Chromosomal Location of the Transgene Insertions Arrows indicate the insertion sites of *P[wFl-ocn-lacZ]* (black) and *P[YEStes-lacZ]* (gray) transgenes as determined by inverse PCR. Nine additional inserts could be assigned only to the X chromosome or autosomes by genetic crosses and are not shown.

Could our results be explained by something other than male germline X inactivation? One possibility is that there is an insertional bias of our transgenes that differs between the X chromosome and the autosomes. For example, X-linked inserts could preferentially target inactive or heterochromatic regions. To investigate this possibility, we used inverse PCR to map the insertion sites (Figure 5). We found that the insertions span the euchromatic regions of the X and autosomes, with many being in or near genes (Table S1). Thus, our mapping results run counter to the expectations of insertional bias as a cause of the observed differences in expression. Another possibility is that insertion of the transgenes onto the X chromosome may cause rearrangements or other disruptions to the gene or promoter that prevent proper expression. However, by remobilizing multiple, independent X inserts to new autosomal locations, we have shown that their expression can be restored. Thus, the X-linked insertions must have been intact. Finally, a lack of proper dosage compensation of transgenes inserted onto the X chromosome could possibly lead to reduced expression. We consider this mechanism unlikely for two reasons. First, X chromosome dosage compensation has been shown to occur on a global level in the *Drosophila* germline [33]. Second, the expression assays for the autosomal-insert lines were performed on flies heterozygous for the insertion. Thus, even if dosage compensation did not occur, we would expect to observe equal expression of X-linked and autosomal transgenes. Any degree of dosage compensation would result in higher activity in the X-insertion lines, which makes our test conservative.

The use of the *ocn* promoter may make our experimental system especially sensitive to the effects of male germline X inactivation for two reasons. First, the promoter fragment used here is rather short (150 bp) and, thus, may be abnormally influenced by differences in chromatin environment between the autosomes and the X chromosome. It should be noted, however, that other known testis-specific promoters are also relatively short, in the range of 76–390 bp [36–38]. Second, *ocn* is likely to be expressed relatively late in spermatogenesis, when the effects of X inactivation should be pronounced. The *ocn* gene was originally identified as one encoding a protein abundant in the testes of mature males, but absent from those of immature males [34]. Our observation that β-galactosidase activity imparted by the *ocn*-*lacZ* transgenes is greatest in proximal regions of the testis (Figure 2) also supports its relatively late expression. Furthermore, levels of β-galactosidase activity, as well as transgene transcript abundance as measured by qRT-PCR, are at least 50-fold lower in the third larval instar stage, when spermatogenesis is not yet complete, than in adult males (unpublished data). Thus, it may be that a large proportion of *ocn* expression occurs after the X chromosome is inactivated. Indeed, if X-linked genes expressed early in spermatogenesis are hypertranscribed through a dosage compensation mechanism [33], the effects of later X inactivation may be masked. Finally, we wish to point out that, although testis-expressed genes are under-represented on the X chromosome, they are not absent. Thus, many X-linked genes involved in spermatogenesis must be expressed at levels sufficient for proper function. This may be a result of their (hyper)transcription early in spermatogenesis. Recently, it has been noted that a region of the X chromosome is enriched for newly evolved, testis-expressed genes [39–41], which suggests that this region may escape germline X inactivation. One of our transgene inserts falls within ∼500 kb of this interval, but does not differ in expression from other X-linked insertions. A higher density of X-linked transgene insertions may reveal specific regions that escape inactivation.

Overall, *P[YEStes-lacZ]* transformants had much lower β-galactosidase activity than *P[wFl-ocn-lacZ]* transformants (Figure 4A). This difference was not observable at the level of mRNA (Figure 4B), suggesting additional regulation at the level of translation. Two major differences between the vectors could account for the discrepancy between the enzymatic assays and the qRT-PCR measurements. The first is the *suppressor of Hairy-wing* chromosomal insulator sequences in *P[YEStes-lacZ]* (Figure 1). However, it seems unlikely that these insulator sequences, which lie far outside of the transcriptional unit, would be involved in post-transcriptional regulation. Furthermore, putting the transgenes into a genetic background homozygous for a mutant *suppressor of Hairy-wing* allele had no effect on levels of β-galactosidase activity (Figure S2). The second difference is that *P[YEStes-lacZ]* contains the *ocn* 3′ untranslated region (UTR) (Figure 1). Although functional information for this 3′ UTR is lacking, the presence of two conserved sequence blocks suggests that it may play a role in the regulation of expression (Figure S1).

Our finding that a testis-specific gene is not properly expressed when located on the X chromosome provides compelling experimental evidence for male germline X inactivation in *Drosophila*, something that was first proposed over thirty years ago [27]. It is also consistent with a selective explanation for the overabundance of retrotransposed genes that have moved from the X to the autosomes [8]. If such genes have a beneficial effect when expressed in testis (especially in later stages of spermatogenesis), then selection would favor the maintenance of autosomal copies. The acquisition of expression late in spermatogenesis may even predispose a gene to adaptive evolution, as testis-expressed genes appear to be targets of positive selection more often than genes of other expression classes [42]. Our results also have relevance to the SAXI hypothesis [23], which proposes that sexual antagonism leads to the selective relocation of male-beneficial genes expressed late in spermatogenesis to the autosomes. After all such genes have been relocated, selection could favor global inactivation of the X chromosome during spermatogenesis to prevent the expression of female-beneficial genes that have a harmful effect when expressed in males. Alternatively, the X may be inactive at this stage simply because it no longer contains genes with the proper regulatory sequences required for male germline expression. Our results are consistent with the former scenario, as the *ocn* promoter, which drives testis-specific expression on autosomes, does not function properly when relocated to the X chromosome.

## Materials and Methods

### Transformation vector construction.

Two different expression vectors that combined the *ocn* promoter of D. melanogaster with the *lacZ* coding region of E. coli were generated using standard techniques [43]. For the first, we PCR-amplified a 150-bp fragment of D. melanogaster genomic DNA that spanned bases 25,863,383–25,863,532 of Chromosome 3R in genome release 5 (http://www.fruitfly.org/sequence/release5genomic.shtml). The amplified region includes 80 bases of 5′ flanking sequence and 70 bases of 5′ UTR of the *ocn* gene, corresponding to bases −165 to −16 relative to the A in the ATG start codon. We chose to end the promoter fragment at −16 because the preceeding sequence presented a good target for PCR-primer design; we know of no functional reason to include or exclude the final 15 bp before the start codon. The PCR product was cloned directly into the pCR2.1-TOPO vector (Invitrogen, http://www.invitrogen.com/). The identity and orientation of the cloned fragment were confirmed by restriction analysis. A 3.5-kb NotI fragment containing the complete *E. coli lacZ* open reading frame was excised from the plasmid pCMV-SPORT-βgal (Invitrogen) and inserted into the NotI site of the above plasmid, just downstream of the *ocn* promoter and in the same orientation. A 3.6-kb fragment containing the *ocn* promoter and the *lacZ* coding region was then excised as an SpeI/XbaI fragment and cloned into the SpeI site of the p*P[wFl]* transformation vector. This vector is based on the *P* transposable element and contains the *D. melanogaster white (w)* gene as a selectable marker [44]. The final construct was designated p*P[wFl-ocn-lacZ]* (Figure 1A).

The second expression vector contained the *ocn* promoter described above as well as the *ocn* 3′ UTR sequence (Figure S1). The *ocn* promoter was excised from the pCR2.1-TOPO vector as a BamHI/XbaI fragment and inserted into the BamHI/XbaI sites of the plasmid pUC18 (Invitrogen). The *ocn* 3′ UTR sequence was PCR-amplified from genomic DNA corresponding to bases 25,862,721–25,862,830 of chromosome arm 3R (bases −16 to +93 relative to the T in the TGA stop codon) and cloned into the pCR2.1-TOPO vector. After confirming the identity and orientation of the cloned fragment by restriction analysis, a HindIII fragment (where one HindIII site was internal to the 3′ UTR fragment, occurring 30 bp from the 5′ end) was extracted and inserted into the HindIII site of the pUC18 plasmid containing the *ocn* promoter, such that the promoter and 3′ UTR were in the same orientation. An SpeI fragment containing both the promoter and the 3′ UTR was then excised and cloned into the XbaI site of the YES vector [35]. This vector is also based on the *P* transposable element and contains the *yellow (y)* gene of D. melanogaster as a selectable marker. Additionally, it contains binding sites for the Suppressor of Hairy-wing protein that flank the inserted DNA and serve to insulate it from position effects caused by random insertion of the vector into the genome [35]. The resulting transformation vector was designated as YEStes (YES vector for testes-specific expression) and contains the *ocn* promoter and 3′ UTR separated by unique XbaI and NotI restriction sites. To complete the expression construct, a 3.5-kb NotI fragment of the plasmid pCMV-SPORT-βgal containing the complete *lacZ* open reading frame was cloned into the NotI site of the YEStes vector in the appropriate orientation. This final construct was designated p*P[YEStes-lacZ]* (Figure 1B).

### Germline transformation.

Plasmid DNA of the above expression constructs was purified using the QIAprep Spin kit (QIAGEN, http://www.qiagen.com/) and used for microinjection of early stage embryos of the *y w;* Δ2–3, *Sb*/TM6 strain of D. melanogaster following standard procedures [45,46]. Because it carries both the *y* and *w* mutations, this strain could be used for both transformation vectors. The Δ2–3 *P* element on the third chromosome served as source of transposase [47]. Following transformation, all lines were crossed to a *y w* stock to remove the transposase source.

In cases where the transgene insertion was linked to the Δ2–3 source of transposase, the inserts were immediately remobilized by crossing transformed males to *y w* females and selecting offspring carrying the transgene, but not the Δ2–3 element. These flies were then mated to *y w* flies of the opposite sex to establish stable transgenic lines.

X-linked insertions were identified by crossing transformed males to *y w* females and following inheritance of the phenotypic marker (*y* ^+^ or *w* ^+^); crosses in which all daughters, but no sons, showed the marker phenotype revealed X linkage. Some X-linked insertions were mobilized to the autosomes by the following procedure. Transformed females were mated to *y w;* Δ2–3, *Sb*/TM6 males and the male offspring carrying both the transgene and the Δ2–3 source of transposase were mated to *y w* females. From this cross, we selected male offspring carrying the transgene (which could not be on the X chromosome inherited from the mother). These males were mated to *y w* females to establish stable transformed lines with new autosomal insertions of the transgene.

To map the intrachromosomal location of the transgene insertions, the genomic sequence flanking the *P*-element vector was determined by sequencing the products of inverse PCR [48]. Briefly, genomic DNA was extracted from insertion-bearing flies and digested with either HpaII or HinP1I. The digestion products were self-ligated and used as a template for PCR with primer pairs Pry1 (5′-CCTTAGCATGTCCGTGGGGTTTGAAT-3′) and Pry2 (5′-CTTGCCGACGGGACCACCTTATGTTATT-3′); Plac1 (5′-CACCCAAGGCTCTGCTCCCACAAT-3′) and Plac4 (5′-ACTGTGCGTTAGGTCCTGTTCATTGTT-3′) to determine 3′ or 5′ flanking sequences, respectively. PCR products were sequenced with BigDye v1.1 chemistry on a 3730 automated sequencer (Applied Biosystems, http://www.appliedbiosystems.com/) using the PCR primers as sequencing primers. In all cases, the chromosomal locations assigned by inverse PCR were consistent with those determined by genetic crosses.

### **β**-Galactosidase assays.

To determine in vivo expression levels of our transgenic constructs, we measured the level of β-galactosidase activity in transformed flies. For all autosomal insert lines, transformed males were mated to *y w* females and offspring heterozygous for the transgene insertion were used for assays. For transformants with X-linked inserts, females were mated to *y w* males and offspring heterozygous (female) or hemizygous (male) for the transgene insertion were used for assays. In all cases, the offspring were collected shortly after eclosion and separated by sex until they were assayed at age 5–7 d. All flies were raised on cornmeal-molasses medium at 25 °C.

For assays of β-galactosidase activity, five adult flies of the same sex were homogenized in 150 μl of a buffer containing 0.1 M Tris-HCl, 1 mM EDTA, and 7 mM 2-mercaptoethanol at pH 7.5. After incubation on ice for 15 min, the homogenates were centrifuged at 12,000 *g* for 15 min at 4 °C and the supernatant containing soluble proteins was retained. For each assay, 50 μl of this supernatant were combined with 50 μl of assay buffer (200 mM sodium phosphate [pH 7.3], 2 mM MgCl_2_, 100 mM 2-mecaptoethanol) containing 1.33 mg/ml *o*-nitrophenyl-β-D-galactopyranoside. β-Galactosidase activity was measured by following the change in absorbance at 420 nm over 30 min at 25 °C. β-Galactosidase activity units were quantified as the change in absorbance per minute multiplied by 1,000 (mOD/min). For all transformed lines, we performed at least two technical and two biological replicates (always in equal numbers), where the former used the same soluble protein extraction and the latter used extractions from independent cohorts of flies. The activity of each line was calculated as the mean over all replicates, with the variance and standard error calculated among replicates. For comparisons between chromosomes or vectors, we averaged over the means of the individual lines and used the among-line variation to calculate variance, standard error, and CV. This approach is conservative, as the among-line differences (position effects) tended to be the largest source of variation. Statistical tests for differences between groups were performed using nonparametric methods, such as the Mann-Whitney U test, that do not rely on estimates of variance. For our purposes this approach is conservative.

For lines that showed β-galactosidase activity in adult males, we also performed assays on gonadectomized males. This was done following the above protocol, after removal of the testes by manual dissection. For visualizing β-galactosidase activity in whole tissues, we incubated dissected testes in the above assay buffer containing 1 mg/ml ferric ammonium citrate and 1.8 mg/ml of S-GAL sodium salt (Sigma-Aldrich, http://www.sigmaaldrich.com/) for 6 h at 37 °C.

### Quantitative reverse-transcription PCR (qRT-PCR).

To measure expression at the level of transcription (mRNA abundance), we performed qRT-PCR using a TaqMan assay (Applied Biosystems) designed specifically to our transgene (i.e., spanning the junction between the *ocn* 5′ UTR and the *lacZ* coding region). For this, 1 μg of DNase I-treated total RNA isolated from heterozygous (autosomal insertions) or hemizygous (X insertions) males was reverse transcribed using Superscript II reverse transcriptase and random hexamer primers (Invitrogen) according to the manufacturer's protocol. A 1:10 dilution of the resulting cDNA was used as template for PCR on a 7500 Fast Real-Time PCR System (Applied Biosystems). The average threshold cycle value (Ct) was calculated from two technical replicates per sample. Expression of the transgene was standardized relative to the ribosomal protein gene *RpL32* (TaqMan probe ID Dm02151827). Relative expression values were determined by the ΔΔCt method according the formula 2^−(ΔCt*x* − ΔCt*min*)^, where ΔCt*_x_* = Ct*_transgene_* − Ct*_RpL32_* for a given transformed line *x*, and ΔCt*_min_* represents the corresponding value of the line displaying the lowest level of transgene relative to *RpL32* expression. Statistical analyses were performed as described above for β-galactosidase activity.

## Supporting Information

Figure S1 Sequence Alignment of the *ocn* Promoter and 3′ UTR (A) Alignment of the *ocn* 5′ flanking and 5′ UTR sequences of *D. melanogaster, D. simulans, D. sechellia, D. yakuba,* and D. erecta. The arrowheads indicate the boundaries of the *ocn* promoter sequence included in our expression constructs. The transcriptional start site is indicated by an arrow.
(B) Alignment of the *ocn* 3′ UTR sequences of *D. melanogaster, D. simulans, D. sechellia, D. yakuba,* and D. erecta. The two conserved regions are shaded. The arrowheads indicate the boundaries of the 3′ UTR sequence included in our expression construct.
(40 KB PDF)

Figure S2 Effect of *suppressor of Hairy-wing* Genetic Background on Transgene Expression The β-galactosidase activity imparted by the transgenes was measured in a background where the third chromosome was homozygous for either the mutant *su(Hw)^8^* allele (solid bars) or the wild-type allele (open bars).
(A) Activity comparison of eight heterozygous second-chromosomal insertions.
(B) Activity comparison of eight hemizygous X-chromosomal insertions. In both cases, the genetic background had no significant effect on activity (two-tailed Wilcoxon signed ranks test, *p* > 0.10). Error bars indicate the standard error of the mean, calculated from the variance among all replicate measurements within each independent insertion line.
(65 KB PDF)

Table S1 Chromosomal Locations of Transgene Insertions (25 KB PDF)

### Accession Numbers

The FlyBase (http://www.flybase.org/) accession numbers for the genes discussed in this article are *janus B* (CG7931; FBgn0001281), *ocnus* (CG7929; FBgn0041102), *RpL32* (CG7939; FBgn0002626), *white* (CG2759; FBgn0003996), and *yellow* (CG3757; FBgn0004034).

## Abbreviations

CV: coefficient of variation
qRT-PCR: quantitative reverse-transcription PCR
SAXI: sexually antagonistic X inactivation
UTR: untranslated region

## References

[pbio-0050273-b001] Charlesworth B (1996). The evolution of chromosomal sex determination and dosage compensation. Curr Biol.

[pbio-0050273-b002] Rice WR (1996). Evolution of the Y sex chromosome in animals. Bioscience.

[pbio-0050273-b003] Ganguly R, Swanson KD, Ray K, Krishnan R (1992). A BamHI repeat element is predominantly associated with the degenerating neo-Y chromosome of Drosophila miranda but absent in the Drosophila melanogaster genome. Proc Natl Acad Sci U S A.

[pbio-0050273-b004] Steinemann M, Steinemann S (2000). Common mechanisms of Y chromosome evolution. Genetica.

[pbio-0050273-b005] Steinemann S, Steinemann M (2001). Biased distribution of repetitive elements: A landmark for neo-Y chromosome evolution in Drosophila miranda. Cytogenet Cell Genet.

[pbio-0050273-b006] Bachtrog D (2005). Sex chromosome evolution: Molecular aspects of Y-chromosome degeneration in *Drosophila*. Genome Res.

[pbio-0050273-b007] Vicoso B, Charlesworth B (2006). Evolution on the X chromosome: Unusual patterns and processes. Nat Rev Genet.

[pbio-0050273-b008] Betrán E, Thornton K, Long M (2002). Retroposed new genes out of the X in *Drosophila*. Genome Res.

[pbio-0050273-b009] Betrán E, Long M (2003). Dntf-2r, a young *Drosophila* retroposed gene with specific male expression under positive Darwinian selection. Genetics.

[pbio-0050273-b010] Betrán E, Bai Y, Motiwale M (2006). Fast protein evolution and germ line expression of a *Drosophila* parental gene and its young retroposed paralog. Mol Biol Evol.

[pbio-0050273-b011] Kalamegham R, Sturgill D, Siegfried E, Oliver B (2006). Drosophila *mojoless,* a retroposed *gsk-3,* has functionally diverged to acquire an essential role in male fertility. Mol Biol Evol.

[pbio-0050273-b012] Parisi M, Nuttall R, Naiman D, Bouffard G, Malley J (2003). Paucity of genes on the *Drosophila* X chromosome showing male-biased expression. Science.

[pbio-0050273-b013] Ranz JM, Castillo-Davis CI, Meiklejohn CD, Hartl DL (2003). Sex-dependent gene expression and evolution of the *Drosophila* transcriptome. Science.

[pbio-0050273-b014] Hambuch TM, Parsch J (2005). Patterns of synonymous codon usage in Drosophila melanogaster genes with sex-biased expression. Genetics.

[pbio-0050273-b015] Connallon T, Knowles LL (2005). Intergenomic conflict revealed by patterns of sex-biased gene expression. Trends Genet.

[pbio-0050273-b016] Rogers DW, Carr M, Pomiankowski A (2003). Male genes: X-pelled or X-cluded?. Bioessays.

[pbio-0050273-b017] Schlotterer C (2003). Where do male genes live?. Science.

[pbio-0050273-b018] Oliver B, Parisi M (2004). Battle of the Xs. Bioessays.

[pbio-0050273-b019] Dorus S, Busby SA, Gerike U, Shabanowitz J, Hunt DF, Karr TL (2006). Genomic and functional evolution of the Drosophila melanogaster sperm proteome. Nat Genet.

[pbio-0050273-b020] Swanson WJ, Clark AG, Waldrip-Dail HM, Wolfner MF, Aquadro CF (2001). Evolutionary EST analysis identifies rapidly evolving male reproductive proteins in *Drosophila*. Proc Natl Acad Sci U S A.

[pbio-0050273-b021] Rice WR (1984). Sex chromosomes and the evolution of sexual dimorphism. Evolution.

[pbio-0050273-b022] Charlesworth B, Coyne JA, Barton NH (1987). The relative rates of evolution of sex chromosomes and autosomes. Am Nat.

[pbio-0050273-b023] Wu CI, Xu EY (2003). Sexual antagonism and X inactivation—The SAXI hypothesis. Trends Genet.

[pbio-0050273-b024] Richler C, Soreq H, Wahrman J (1992). X inactivation in mammalian testis is correlated with inactive X-specific transcription. Nat Genet.

[pbio-0050273-b025] Kelly WG, Schaner CE, Dernburg AF, Lee MH, Kim SK (2002). X-chromosome silencing in the germline of C. elegans. Development.

[pbio-0050273-b026] Fong Y, Bender L, Wang W, Strome S (2002). Regulation of the different chromatin states of autosomes and X chromosomes in the germ line of C. elegans. Science.

[pbio-0050273-b027] Lifschytz E, Lindsley DL (1972). The role of X-chromosome inactivation during spermatogenesis. Proc Natl Acad Sci U S A.

[pbio-0050273-b028] McKee BD, Handel MA (1993). Sex chromosomes, recombination, and chromatin conformation. Chromosoma.

[pbio-0050273-b029] Hoyle HD, Hutchens JA, Turner FR, Raff EC (1995). Regulation of beta-tubulin function and expression in *Drosophila* spermatogenesis. Dev Genet.

[pbio-0050273-b030] Rastelli L, Kuroda MI (1998). An analysis of *maleless* and histone H4 acetylation in Drosophila melanogaster spermatogenesis. Mech Dev.

[pbio-0050273-b031] Gu W, Szauter P, Lucchesi JC (1998). Targeting of MOF, a putative histone acetyl transferase, to the X chromosome of Drosophila melanogaster. Dev Genet.

[pbio-0050273-b032] Smith ER, Pannuti A, Gu W, Steurnagel A, Cook RG (2000). The *Drosophila* MSL complex acetylates histone H4 at lysine 16, a chromatin modification linked to dosage compensation. Mol Cell Biol.

[pbio-0050273-b033] Gupta V, Parisi M, Sturgill D, Nuttall R, Doctolero M (2006). Global analysis of X-chromosome dosage compensation. J Biol.

[pbio-0050273-b034] Parsch J, Meiklejohn CD, Hauschteck-Jungen E, Hunziker P, Hartl DL (2001). Molecular evolution of the *ocnus* and *janus* genes in the Drosophila melanogaster species subgroup. Mol Biol Evol.

[pbio-0050273-b035] Patton JS, Gomes XV, Geyer PK (1992). Position-independent germline transformation in *Drosophila* using a cuticle pigmentation gene as a selectable marker. Nucleic Acids Res.

[pbio-0050273-b036] Michiels F, Gasch A, Kaltschmidt B, Renkawitz-Pohl R (1989). A 14 bp promoter element directs the testis specificity of the *Drosophila* beta 2 tubulin gene. EMBO J.

[pbio-0050273-b037] Yanicostas C, Lepesant JA (1990). Transcriptional and translational *cis*-regulatory sequences of the spermatocyte-specific *Drosophila janusB* gene are located in the 3′ exonic region of the overlapping *janusA* gene. Mol Gen Genet.

[pbio-0050273-b038] Nurminsky DI, Nurminskaya MV, De Aguiar D, Hartl DL (1998). Selective sweep of a newly evolved sperm-specific gene in *Drosophila*. Nature.

[pbio-0050273-b039] Levine MT, Jones CD, Kern AD, Lindfors HA, Begun DJ (2006). Novel genes derived from noncoding DNA in Drosophila melanogaster are frequently X-linked and exhibit testis-biased expression. Proc Natl Acad Sci U S A.

[pbio-0050273-b040] Begun DJ, Lindfors HA, Kern AD, Jones CD (2007). Evidence for *de novo* evolution of testis-expressed genes in the Drosophila yakuba/Drosophila erecta clade. Genetics.

[pbio-0050273-b041] Chen ST, Cheng HC, Barbash DA, Yang HP (2007). Evolution of *hydra*, a recently evolved testis-expressed gene with nine alternative first exons in Drosophila melanogaster. PLoS Genet.

[pbio-0050273-b042] Pröschel M, Zhang Z, Parsch J (2006). Widespread adaptive evolution of *Drosophila* genes with sex-biased expression. Genetics.

[pbio-0050273-b043] Sambrook J, Fritsch EF, Maniatis T (1989). Molecular cloning: A laboratory manual. Ed. 2.

[pbio-0050273-b044] Siegal ML, Hartl DL (1996). Transgene coplacement and high efficiency site-specific recombination with the Cre/*loxP* system in Drosophila. Genetics.

[pbio-0050273-b045] Spradling AC, Rubin GM (1982). Transposition of cloned P-elements into *Drosophila* germ line chromosomes. Science.

[pbio-0050273-b046] Rubin GM, Spradling AC (1982). Genetic transformation of *Drosophila* with transposable element vectors. Science.

[pbio-0050273-b047] Robertson HM, Preston CR, Phillis RW, Johnson-Schlitz DM, Benz WK (1988). A stable genomic source of *P* element transposase in Drosophila melanogaster. Genetics.

[pbio-0050273-b048] Bellen HJ, Levis RW, Liao G, He Y, Carlson JW (2004). The *BDGP* gene disruption project: Single transposon insertions associated with 40% of *Drosophila* genes. Genetics.

