# Supplementary material for: X Chromosome Inactivation during Drosophila Spermatogenesis
Source: PLoS Biol. 2007 Oct 9;5(10):e273. doi: 10.1371/journal.pbio.0050273 (PMC2001211; doi:10.1371/journal.pbio.0050273)
Supplement: Supplementary file 1 [file pbio.0050273.sg001.pdf]

A

```
Dmel ACTTTTTGAATGATCACATGTGCTCCGATTTTCGTGTCAGTCTTGAATTTAGCATACGACCGGAAGGCTTCTGACAAAATCAGTCTA
Dsim ACTTTTTGAACGATCACATGTGCTCCGATTTTCGTGTCAGTATAGAATTTATTATACGACCGGACGGCTTCTGACAAAATCAGTCTA
Dsec ACTTTTTAAACGATCACATGTGCTCCGATTTTTGTGCCAGTCTTGAATTTAGCATACGACCGGAAGGCTTCTGACAAAATCAGTCTA
Dyak ACTTTTTTAATCATCACATGTGCCACGATTTTTGTGTCAGTTTCGAATTTGGCATACGACCTGAAGGCTTCTGATGAAATCAGTCTA
Dere ACTTTTTAAATGATCACATGTGCTACGATTTTCGTGTCAGCTTTCAATTTAGCATACGATCCGAGGGCTTCCAATGAAATCAGTCTA

Dmel GCTGGAGCCTTGCTACCGTTCATACCAGTTTCTTTTCGGAATATTTCATCTAAATTCCAGTGCGTTTTCCT-AAAATTTTTCCAAA
Dsim GCTGGAGCCCTGCTACCGTTCAGACCAGTTTCTTTTCGGAATATTTCATCTAAATTCCAGTGCGTTTTCCT-AAAATTTTTCCAAA
Dsec GCTGGAGCCTTGCTACCGTTCAGACCAGTTTCTTTTCGGAATATTTCATCTAAATTCCAGTGCGTTTTCCT-AAAAATTTTTCAAA
Dyak GCTGGAGCCTTGCTACCGTTCGGACCAGATTCTTTTCGGAATATTTCATCTAAATTCCAGTGCCTTTTTCTTAAAATTTTTCCAAA
Dere GCTGGGTCCTTGCTACCGTTCAGACCAGTTTCTTTTCGGAATATTTCATCTAAATTCCAGTGCCTTTTTCTTAAAATTTTTCCAAA
```

B

```
Dmel TGAAGGCGTTTGAAAGCTTGTTTTATTTTTCCTAACTGCCCTGTTGGCAATTTATAATACAACAAAAAA-TTAGTAAATACATGGCTACATATCGATTT
Dsim TGAAGGCGCTTAAAGGCTTGTTTTATTTTTCCTAACTGCCCTGTTGGCAATTTATAATACAAAAAAA---TAAGTAAATACATGGCTACATATCGATTC
Dsec TGAAGGCGCTTAAAAGCTTGTTTTATTTTTCTTAACTGCCCTGTTGGCAATTTATAATACAAAAAAAAAATATGTAAATACATGGCTACATATCGATTC
Dyak TGAAGGCGTTTT-AAGCTTGTTTAATTTTTC--AA-TGCCCTGTTGGCAATATATTATATAAAAAAA-CAA-AGTAAATACATGGCTAAATATC-ATTC
Dere TGAATGCGTTTTAAAGCTTGTTTAATTTTTCAAAGCTGCCCTGTTGGCAATTTCTTATATAAAAAATACAA-AGTAAATACATGGCTAAATATC-ATTC
```

Figure S1. (A) Alignment of the *ocn* 5' flanking and 5' UTR sequences of *D. melanogaster*, *D. simulans*, *D. sechellia*, *D. yakuba*, and *D. erecta*. The arrowheads indicate the boundaries of the *ocn* promoter sequence included in our expression constructs. The transcriptional start site is indicated by an arrow. (B) Alignment of the *ocn* 3' UTR sequences of *D. melanogaster*, *D. simulans*, *D. sechellia*, *D. yakuba*, and *D. erecta*. The two conserved regions are shaded. The arrowheads indicate the boundaries of the 3' UTR sequence included in our expression construct.
