# Supplementary material for: X Chromosome Inactivation during Drosophila Spermatogenesis
Source: PLoS Biol. 2007 Oct 9;5(10):e273. doi: 10.1371/journal.pbio.0050273 (PMC2001211; doi:10.1371/journal.pbio.0050273)
Supplement: Supplementary file 2 [file pbio.0050273.sg002.pdf]

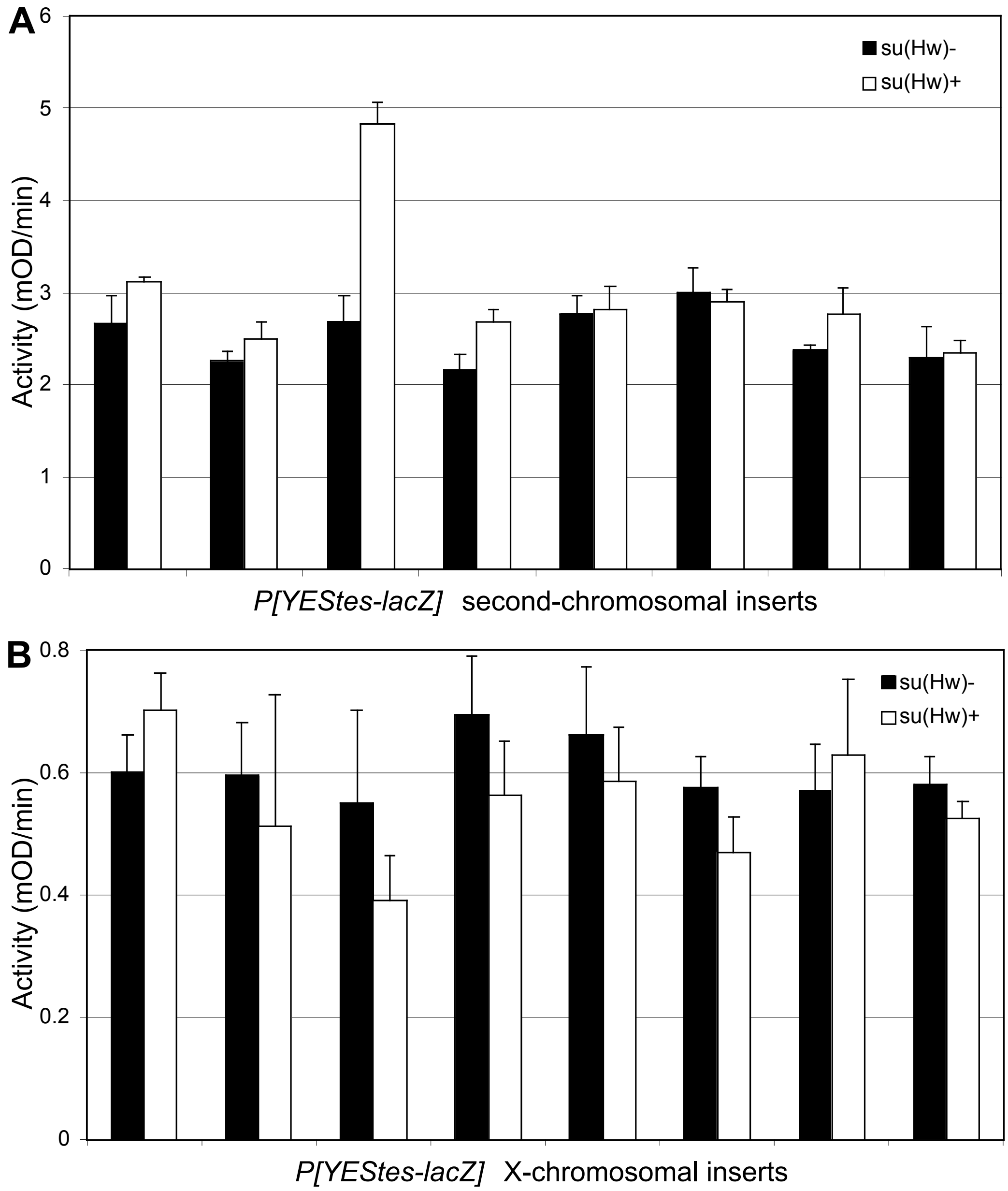

Figure S2. The effect of *suppressor of Hairy-wing* genetic background on *P[YEStes-lacZ]* transgene expression in adult males. The β-galactosidase activity imparted by the transgenes was measured in a background where the third chromosome was homozygous for either the mutant *su(Hw)*^{8} allele (solid bars) or the wild-type allele (open bars). (A) Activity comparison of eight heterozygous second-chromosomal insertions. (B) Activity comparison of eight hemizygous X-chromosomal insertions. In both cases, the genetic background had no significant effect on activity (two-tailed Wilcoxon signed ranks test, $P > 0.10$). Error bars indicate the standard error of the mean, calculated from the variance among all replicate measurements within each independent insertion line.
