# Supplementary material for: X Chromosome Inactivation during Drosophila Spermatogenesis
Source: PLoS Biol. 2007 Oct 9;5(10):e273. doi: 10.1371/journal.pbio.0050273 (PMC2001211; doi:10.1371/journal.pbio.0050273)
Supplement: Supplementary file 3 [file pbio.0050273.st001.pdf]

Table S1: Chromosomal locations of transgene insertions

Line	Chrom	Cyt Band	Coordinate (v5.1)	Location	Comment	Proximal gene	Distal gene
wol12X	X	7B1	7231447*	intergenic		CG18155	CG1435
wol21X	X	10E3	11699401	CG4147	in exon		
wol13X	X	11E3	13101216	CG1903	in intron		
wol23X	X	19F1	20994197	intergenic		CG15445	CG34120
wol24X	X	10E3	11687344*	CG15224	in intron		
wol20X	X	15A7	16677891*	intergenic		CG9623	CG4742
wol19X	X	16A1	17197389*	CG5445	in exon		
wol25X	X	n.m.					
wol5X	X	n.m.					
wol4	2L	27F4	7423613	intergenic		CG5261	CG5229
wol7	2R	42C6	2603250	CG3409	in exon		
wol9	2R	56E1	15518667*	CG9218	in exon		
wol11	3L	61C9	749342	intergenic		CG13897	CG1007
wol6	3L	66C12	8414592	intergenic		CG32354	CG7037
wol18	3L	70F4	14751002*	CG33261	in exon		
wol16	3L	79A2	21872663	intergenic		CG14563	CG7437
wol2	3R	82E4	790802*	heterochrom			
wol1	3R	84B1	2799036*	intergenic		CG41463	CG41464
wol14	3R	85F10	5920571*	intergenic		CG5361	CG6203
wol3	3R	89E11	12882012	CG5201	in intron		
wol15	3R	91D4	14743978	CG17836	in exon		
wol17	3R	91F4	14983880*	CG11779	in intron		
wol10	2	n.m.					
wol8	2	n.m.					
ylz22X	X	4F9	5312216	CG3249	in intron		
ylz9X	X	5A12	5574020	intergenic		CG3171	CG15779
ylz20X	X	11E1	13022326	CG32368	in exon		
ylz15X	X	14A1	15834425	CG9126	in intron		
ylz19X	X	16A1	17195978	CG8649	in exon		
ylz18X	X	16A1	17196628	CG5445	in exon		
ylz17X	X	16B10	17552922	CG5870	in intron		
ylz16X	X	n.m.					
ylz21X	X	n.m.					
ylz23X	X	n.m.					
ylz6	2L	23A3	2753160	CG9894	in intron		
ylz4	2L	24C2	3730445	intergenic		CG2822	CG10019
ylz11	2R	41F9	1642051	CG12792	in exon		
ylz10	2R	42C3	2549792	CG15845	in exon		
ylz5	2R	43E16	3670803	CG1555	in exon		
ylz7	2R	50A13	9389601	CG6033	in exon		
ylz3	2R	53F8	12984754	CG8938	in intron		
ylz12	3L	61C9	749342	intergenic		CG13897	CG1007
ylz13	3L	66D8	8609567	CG6282	in exon		
ylz8	3L	76C5	19784609	CG8742	in exon		
ylz1	2	n.m.					
ylz2	3	n.m.					

wol = *P[wFl-ocn-lacZ]* , ylz = *P[YEStes-lacZ]*

n.m. = not mapped by inverse PCR

* = approximate location, precise insertion site not obtained
